# Splenic arteriovenous fistula leading to non-cirrhotic portal hypertension: a case report: Splenic arteriovenous fistula leading to non-cirrhotic portal hypertension

**DOI:** 10.1093/gastro/goad015

**Published:** 2023-04-10

**Authors:** Ahmad Abou Yassine, Hassan Al Moussawi, Malek Kreidieh, Loai Dahabra, Mustafa Al-Roubaie, Sanjaya Satapathy

**Affiliations:** Department of Internal Medicine, Staten Island University Hospital, Northwell Health, Staten Island, NY, USA; Division of Gastroenterology, Department of Internal Medicine, Staten Island University Hospital, Northwell Health, Staten Island, NY, USA; Department of Internal Medicine, Staten Island University Hospital, Northwell Health, Staten Island, NY, USA; Department of Internal Medicine, Staten Island University Hospital, Northwell Health, Staten Island, NY, USA; Department of Radiology, North Shore University Hospital, Northwell Health, Manhasset, NY, USA; Department of Gastroenterology, North Shore University Hospital, Northwell Health, Manhasset, NY, USA

## Introduction

Non-cirrhotic portal hypertension (NCPH) is a rare disease characterized by portal hypertension in the absence of cirrhosis or other causes of liver disease and splanchnic venous thrombosis. This case is a rare presentation of NCPH due to traumatic arteriovenous fistula.

## Case presentation

A 54-year-old non-obese male presented to the hospital with 1 day of hematemesis. He had a previous medical history of psoriasis on Humira every other week, ulcerative colitis, and a remote history of exploratory laparotomy secondary to stab wound 15 years ago. Prior to presentation to the emergency department, he had two episodes of hematemesis of fresh blood. He also endorsed loose tarry black stools during the last 2 days, associated with decreased oral intake and lower abdominal discomfort without any pain. For the last 6 months, he noted progressing abdominal distention. In addition, he denied any history of non-steroidal anti-inflammatory drugs/steroid use, history of peptic ulcer disease, alcohol misuse, or family history of liver diseases. Other than Humira injections, he did not take any medications or herbal supplementations. He was diagnosed with ulcerative colitis >20 years ago and was in remission.

**Figure 1.**
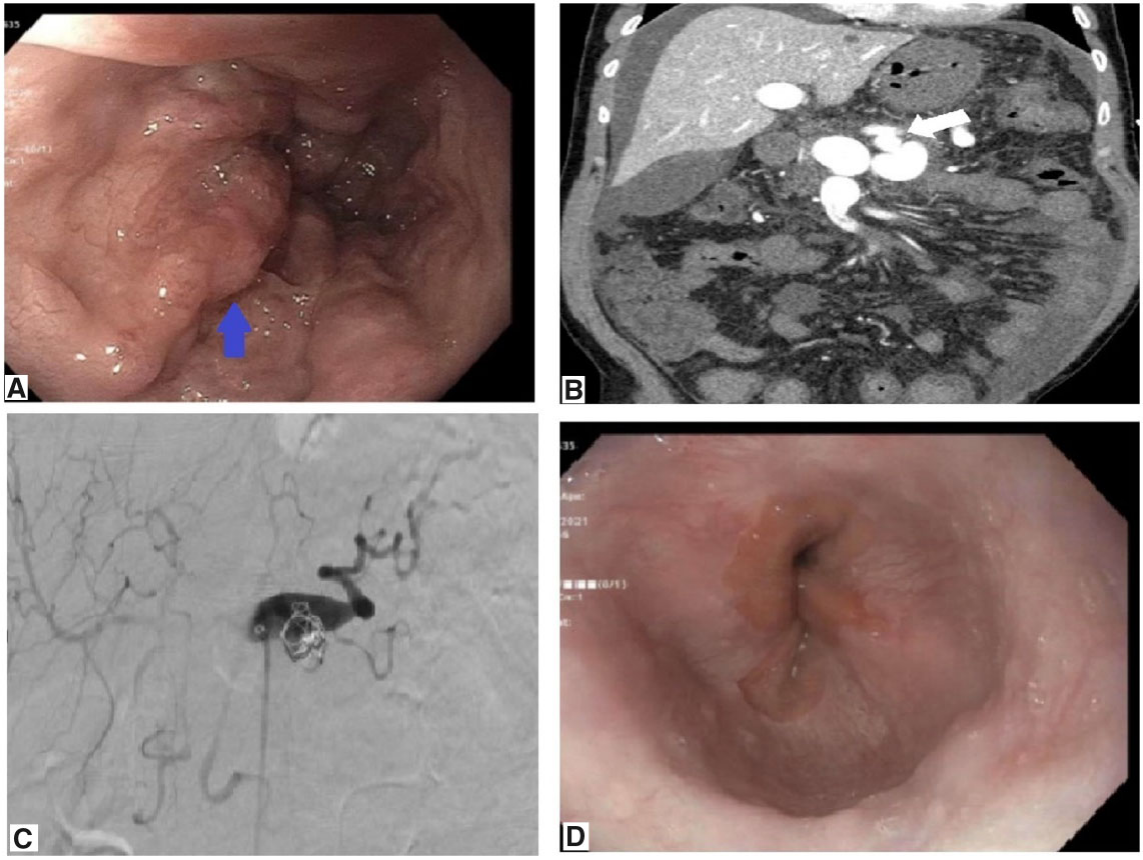
Endoscopy and computed tomography angiography on admission and 2 months later. (A) Endoscopy on initial presentation showing large esophageal varices (blue arrow). (B) Computed tomography angiography on initial presentation showing splenic arteriovenous fistula (white arrow). (C) Percutaneous transcatheter embolization of the arteriovenous fistula and the pseudoaneurysm using 12-mm Amplatzer plug and coil. (D) Repeated endoscopy showing resolution of the esophageal varices after 2 months.

Upon admission, the patient was found to be tachycardic with a heart rate of 135 beats per minute, otherwise his vitals were within the normal range. He received aggressive fluid resuscitation along with intravenous pantoprazole. His physical examination was pertinent for distended abdomen without tenderness, splenomegaly, or a positive fluid wave test with no signs of hepatic encephalopathy. Also, his rectal examination showed melena. His hemoglobin was 8 g/dl and he did not receive any transfusion. His international normalized ratio was 1.4, platelet 242 × 10^9^/L, alkaline phosphatase 149 U/L, and the results of other liver function tests were normal. Viral serology for hepatitis B and C was negative. Emergently, he underwent esophagogastroduodenoscopy (EGD) that showed large varices (>5 mm) in the middle and distal third of the esophagus and in the fundus of the stomach (IGV1) and changes suggestive of portal hypertensive gastropathy (Figure 1A). Four band ligations were successfully applied and intravenous octreotide was started. Afterward, the patient became hypotensive and required a blood transfusion of 1 unit. Norepinephrine was added and he was admitted to the intensive care unit for stabilization and close monitoring. The results of testing for Wilson disease, hemochromatosis, and autoimmune markers including anti-nuclear antibodies, anti-smooth muscle antibodies, anti-liver kidney microsomal antibodies, anti-mitochondrial antibodies, and alpha-1 antitrypsin were also negative. A subsequent CT angiogram of the abdomen and pelvis showed a fistulous communication between both the splenic artery and vein with pseudoaneurysm, moderate ascites, and diffuse colonic wall thickening with peritoneal stranding (Figure 1B). No radiologic evidence of liver cirrhosis or portal/hepatic vein thrombosis was found. His portal hypertension was explained by the CT angiogram results and the decision for liver biopsy was deferred. Later, he had successful percutaneous transcatheter embolization of both the arteriovenous fistula and the pseudo aneurysm by interventional radiology using a 12-mm Amplatzer plug and coil (Figure 1C). Post embolization angiography showed complete occlusion of the fistula and no arterial flow to the portal venous system. After 2 days of hospitalization and close observation, the patient was discharged home safely on a non-selective beta blocker (Nadolol). At follow-up 2 months later, EGD showed complete resolution of the esophageal and gastric varices with normal-appearing esophagus and fundus (Figure 1D). Nadolol was subsequently stopped. On a follow-up visit at 4 months, Fibro scan was performed and showed F0 score, which mean that the hepatic status of the patient had had a major improvement.

## Discussion

Portal hypertension is a clinical syndrome characterized by gastrointestinal varices, splenomegaly, ascites, and encephalopathy. It is defined by a hepatic vein pressure gradient of >5 mmHg or a portal pressure of >10 mmHg [1]. Liver cirrhosis is the most common etiology causing portal hypertension. NCPH, defined as portal hypertension with preserved liver function, can be caused by multiple etiologies such as chronic infections, immunological disorders, medications or toxins, genetic disorders, and prothrombotic conditions [2]. The most common cause of NCPH in the Western world is schistosomiasis [3], whereas in the eastern world thrombophilia is considered the major etiological factor [4]. Depending on the level of defect in the hepatic circulation, NCPH can be caused by problems on the pre-hepatic, intrahepatic, and post-hepatic level. Many cases of NCPH reported arterio–portal shunts or fistulas on an intrahepatic level but few cases on pre-hepatic shunts were found. Etiologies of these shunts include abdominal trauma, iatrogenic procedures (abdominal surgery, liver biopsy, percutaneous biliary interventions), malignancy, and congenital abnormalities [5, 6].

Patients with NCPH due to arteriovenous fistula can be asymptomatic for a long period of time and may present with life-threatening upper gastrointestinal bleeding, ascites, heart failure, intestinal ischemia, or diarrhea [7, 8]. The accurate diagnosis and effective treatment are critical in preventing further complications of NCPH. Treatment options for splenic arteriovenous fistula include endovascular repair with transvenous embolization and surgical resection with or without splenectomy. The surgical option carries more complication risks such as bleeding and infections. If appropriate, the endovascular repair is preferred over the surgical option given the lower complications of risk and faster recovery time.

In our case, the patient had NCPH due to an arterio–portal pre-hepatic shunt. He presented with massive upper gastrointestinal bleeding 15 years after exploratory laparotomy secondary to a stab wound. These patients usually develop indolent portal hypertension with no other manifestations until they have gastrointestinal bleeding or hepatic encephalopathy. Interestingly, the detection of his splenic arteriovenous fistula followed by percutaneous transcatheter coil embolization led to complete reversion of the conditions and avoided future complications.

NCPH may be challenging to diagnose with the lack of standardized diagnostic criteria. There is no clear algorithm to establish a diagnosis in those patients. Our case sheds light on this entity of NCPH caused by vascular disease and the better outcome with proper diagnosis and treatment.

## Authors’ Contributions

A.A.Y., H.M., M.K., and L.D.: carried out conceptualization, investigation, validation, and writing the original draft of the manuscript. M.A. and S.S.: carried out the validation, writing review, and editing of the manuscript and its critical revision.

## References

[1] Bosch J , Berzigotti A, Garcia-Pagan JC et al The management of portal hypertension: rational basis, available treatments and future options. J Hepatol 2008;48:68–92. 18304681 10.1016/j.jhep.2008.01.021

[2] Schouten JN , Garcia-Pagan JC, Valla DC et al Idiopathic noncirrhotic portal hypertension. Hepatology 2011;54:1071–81. 21574171 10.1002/hep.24422

[3] De Cock KM. Hepatosplenic schistosomiasis: a clinical review. Gut 1986;27:734–45. 3522373 10.1136/gut.27.6.734 PMC1433333

[4] Garcia-Pagán JC , Hernández-Guerra M, Bosch J. Extrahepatic portal vein thrombosis. Semin Liver Dis 2008;28:282–92. 18814081 10.1055/s-0028-1085096

[5] Guzman EA , McCahill LE, Rogers FB. Arterioportal fistulas: introduction of a novel classification with therapeutic implications. J Gastrointest Surg 2006;10:543–50. 16627220 10.1016/j.gassur.2005.06.022

[6] Vauthey JN , Tomczak RJ, Helmberger T et al The arterioportal fistula syndrome: clinicopathologic features, diagnosis, and therapy. Gastroenterology 1997;113:1390–401. 9322535 10.1053/gast.1997.v113.pm9322535

[7] Strodel WE , Eckhauser FE, Lemmer JH et al Presentation and perioperative management of arterioportal fistulas. Arch Surg 1987;122:563–71. 3555408 10.1001/archsurg.1987.01400170069010

[8] Capron JP , Gineston JL, Remond A et al Inferior mesenteric arteriovenous fistula associated with portal hypertension and acute ischemic colitis: successful occlusion by intraarterial embolization with steel coils. Gastroenterology 1984;86:351–5. 6690362

